# Surgical Outcomes of Pancreatic Solid Pseudopapillary Neoplasm: Experiences of 24 Patients in a Single Institute

**DOI:** 10.3390/medicina60060889

**Published:** 2024-05-28

**Authors:** Peng-Yu Ku, Shao-Bin Cheng, Yi-Ju Chen, Chia-Yu Lai, Hsiao-Tien Liu, Wei-Hsin Chen

**Affiliations:** 1 Division of General Surgery, Department of Surgery, Taichung Veterans General Hospital, Taichung 40705, Taiwan; lukephil6@gmail.com (P.-Y.K.);; 2 Division of General Surgery, Department of Surgery, Taichung Tzu Chi Hospital, Taichung 427213, Taiwan

**Keywords:** solid pseudopapillary neoplasm of the pancreas, surgical outcomes, pancreatic endocrine function, pancreatic exocrine function

## Abstract

*Background and Objectives*: The pancreatic solid pseudopapillary neoplasm (SPN), a rare tumor predominantly affecting young women, has seen an increased incidence due to improved imaging and epidemiological knowledge. This study aimed to understand the outcomes of different interventions, possible complications, and associated risk factors. *Materials and Methods*: This study retrospectively analyzed 24 patients who underwent pancreatic surgery for SPNs between September 1998 and July 2020. *Results*: Surgical intervention, typically required for symptomatic cases or pathological confirmation, yielded favorable outcomes with a 5-year survival rate of up to 97%. Despite challenges in standardizing preoperative evaluation and follow-up protocols, aggressive complete resection showed promising long-term survival and good oncological outcomes. Notably, no significant differences were found between conventional and minimally invasive (MI) surgery in perioperative outcomes. Histopathological correlations were lacking in prognosis and locations. Among the patients, one developed diffuse liver metastases 41 months postoperatively but responded well to chemotherapy and transcatheter arterial chemoembolization, with disease stability observed at 159 postoperative months. Another patient developed nonalcoholic steatohepatitis after surgery and underwent liver transplantation, succumbing to poor medication adherence 115 months after surgery. *Conclusions*: These findings underscore the importance of surgical intervention in managing SPNs and suggest the MI approach as a viable option with comparable outcomes to conventional surgery.

## 1. Introduction

Pancreatic solid pseudopapillary neoplasm (SPN) was first described by Frantz in 1959, based on the pathologic examination of three patients [1]. In 1970, Hamoudi and colleagues expanded the literature by adding an additional patient and providing detailed electron microscopic descriptions of the tumor [2]. SPNs are characterized by their gross and histologic appearance, which includes discohesive polygonal cells surrounding delicate blood vessels and forming a solid mass, often with cystic degeneration and intracystic hemorrhage [3]. The neoplastic cells exhibit uniform nuclei, finely stippled chromatin, and nuclear grooves, along with eosinophilic globules, foam cells, and cholesterol clefts [3].

SPNs have also been referred to as solid and papillary tumors, papillary cystic tumors, solid cystic tumors, Hamoudi tumors, or Frantz tumors [4]. Despite these varying names, SPN is the preferred terminology [3]. SPNs are rare, comprising only 1–2% of all pancreatic tumors [4,5]. In 1996, the World Health Organization classified pancreatic SPNs as a borderline malignancy of the exocrine pancreas [6]. The incidence of pancreatic SPNs has increased over the past two decades due to improved imaging techniques and a better epidemiological understanding of the disease [7]. SPNs are most commonly diagnosed in women aged 30–40 years and have a favorable prognosis, with a 5-year survival rate of up to 97% [8,9]. Before the operation, imaging studies were conducted to identify the SPN, utilizing sonography (US), computed tomography (CT), and magnetic resonance imaging (MRI). About one-third of pancreatic SPN cases are located in the pancreatic tail, and another third in the pancreatic head [10]. The benefits of the increasing pre-operative pathology diagnosis of SPN using endoscopic ultrasonography (EUS) have yet to be fully established [11].

Surgical intervention for SPNs was first mentioned by Sanfey and associates in 1983 and is often indicated based on tumor-related symptoms or for pathological diagnosis [12]. Although SPNs are usually localized, 10% to 15% of patients may develop metastases [13,14]. These metastases are often resectable, and complete removal is associated with long-term survival [14,15]. Recent studies have compared the outcomes of conventional distal pancreatectomies with minimally invasive (MI) pancreatic surgeries, including laparoscopic and robot-assisted approaches, which have become increasingly favored [16]. Post-operative oncological outcomes are generally positive, with only 15% of SPN cases progressing to distant metastasis, typically in the liver or peritoneum [10,14].

In our study, we detail our experience in treating pancreatic SPNs, including pre-operative imaging results and pathology diagnoses, procedure comparisons, surgical outcomes, and complications in patients with pathologically confirmed pancreatic SPNs.

## 2. Materials and Methods

### 2.1. Study Group

**Figure 1:**
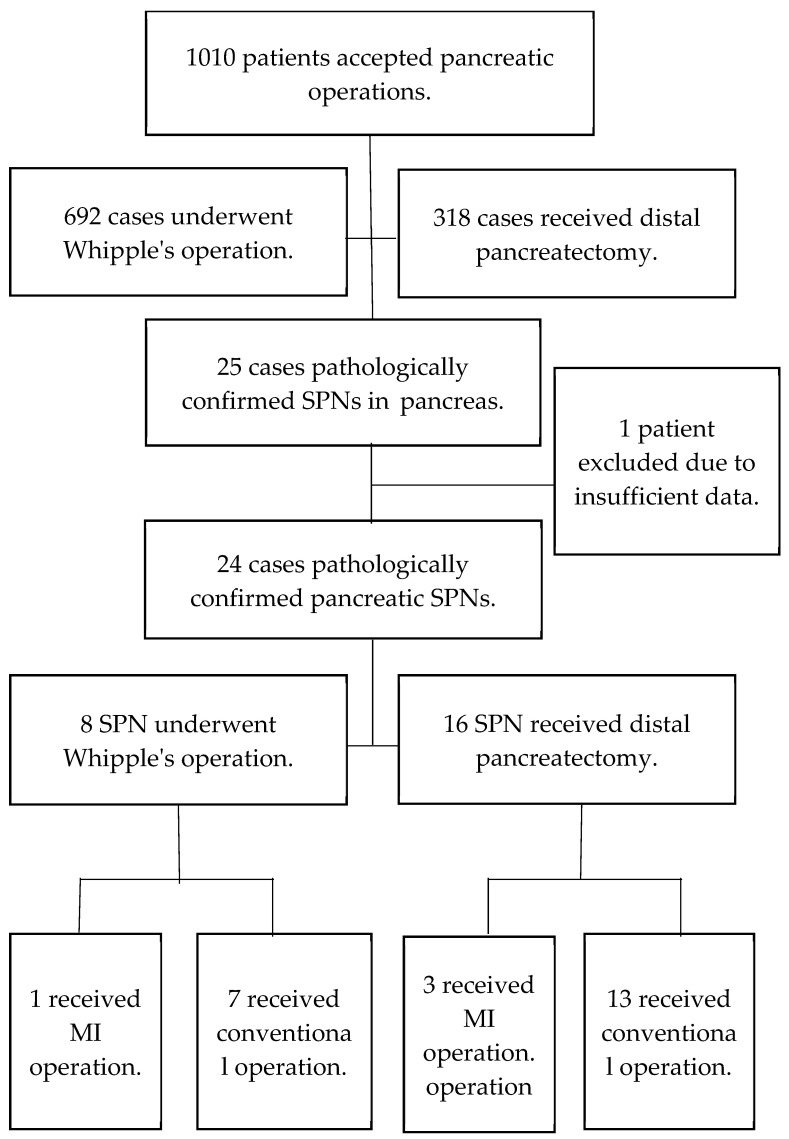
Patient selection flowchart. SPN solid pseudopapillary neoplasm. MI minimal invasive.

We collected the data of patients who underwent pancreatic surgery between September 1998 and July 2020, with a follow-up period ending in March 2023. The patients’ medical records were retrospectively reviewed to identify those who underwent either conventional or MI surgery for pathologically confirmed SPNs in our hospital, a tertiary referral center in Central Taiwan; procedures included pancreaticoduodenectomy (PD), pylorus-preserving pancreaticoduodenectomy (PPPD), partial pancreatectomy (PP), central pancreatectomy (CP), and distal pancreatectomy (DP). A total of 1010 patients underwent pancreatic-related operations, of which 692 underwent PD, and 318 underwent either DP or PP. In total, 25 patients were enrolled in this study according to the pathology report confirming SPN, but 1 patient was excluded due to insufficient data (Figure 1).

### 2.2. Data Collection

We reviewed data, including clinical characteristics, such as sex, age, BMI, and tumor markers; pre-operative imaging studies; and EUS reports. The results of fine needle EUS biopsy for preoperative pathology were analyzed to compare its efficacy with that of preoperative imaging studies.

The surgical outcomes and pathology data analyzed included tumor location and size, status of resection margin, EBL, day of enteral feeding, day of drainage tube removal, and length of hospitalization. The postoperative outcomes were also collected and included complications, postoperative pancreatic fistula, DGE, pathology reports, and post-pancreatectomy diabetes.

### 2.3. Definition of Outcomes

Tumor size was measured and recorded from pathology reports. Blood loss of <50 mL in our hospital was recorded as minimal EBL for the statistical analysis. The amylase concentration in the drainage fluid was exanimated in all the patients, and pancreatic fistulas were graded as follows: biochemical leak, with no clinical impact; grade B, requiring a change in management or adjustment of the clinical approach; and grade C, requiring a major change in clinical management or deviation from the normal clinical approach, according to the Pancreatic Fistula Classification in the International Study Group on Pancreatic Fistula definition [17].

According to the International Study Group of Pancreatic Surgery definition, we also defined DGE according to the duration of nasogastric intubation: 4–7 postoperative days (PODs) as Grade A (mild); 8–14 PODs as Grade B (moderate); and >14 PODs as Grade C (severe) [18].

Post-pancreatectomy diabetes was defined according to American Diabetes Association 2022 guidelines as a fasting plasma sugar of >126 mg/dL or an HbA1c of >6.5% detected during the follow-up period [19].

### 2.4. Statistical Analysis

IBM SPSS version 22.0 (International Business Machines Corp, New York, NY, USA) was used for the statistical analyses. Categorical variables were expressed as percentages, frequencies, or medians with interquartile ranges. Chi-square tests or Mann–Whitney U tests were used to compare categorical data. The correlations between two variables were analyzed with the Pearson correlation coefficient.

### 2.5. Ethics Approval and Consent to Participate

The study adhered to the principles outlined in the Declaration of Helsinki and the International Conference on Harmonisation—Good Clinical Practice guidelines. The study was also reviewed and approved by the Institutional Review Board I & II of Taichung Veterans General Hospital (TCVGH-IRB No.: CE22251A) on 14/06/2022. The need for informed consent was waived by the Institutional Review Board I & II of Taichung Veterans General Hospital.

## 3. Results

### 3.1. λ General Results

Of the 24 patients included in this study, 21 were women, and 3 were men (women-to-men ratio of 7:1) (Table 1). The median age of patients was 34.5 (23.8–41.5) years, with a median body mass index (BMI) of 20.8 (18.9–22.4) kg/m^2^.

Most of our patients were asymptomatic (11/24 cases, 46%), while some of the pancreatic SPNs presented with abdominal pain (8/24 cases, 29%) or palpable abdominal masses (4/24 cases, 17%).

Pancreatic SPNs were predominant in young women in all groups we classified, either by location or procedure (conventional and MI surgery) (Table 1). No statistical differences were identified in terms of age, sex, BMI, size, and tumor marker in comparisons of locations or procedures (Table 1).

Nevertheless, a trend was identified in the procedure comparison; the MI group had a younger age, compared with the conventional group (median, MI 23.5 [19.5–27.5] vs. conventional 38.0 [28.0–45.8] cm; *p* = 0.052).

### 3.2. Preoperative Diagnosis

**Figure 2:**
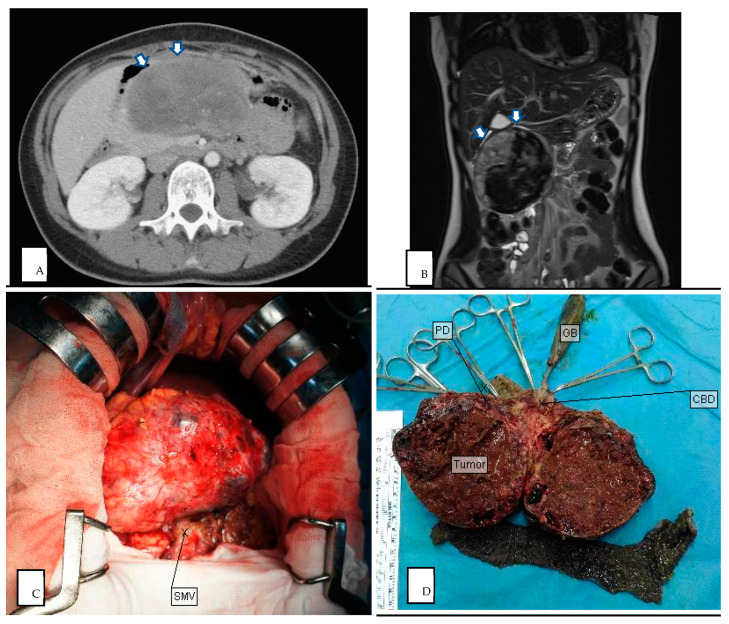
(**A**) Preoperative CT image of SPN in pancreatic head (axial plane, venous phase). (**B**) Preoperative MRI image of SPN in pancreatic tail (coronal plane, T2 weighted). (**C**) Intraoperative photography of SPN in pancreatic head. (**D**) Cross section of intraoperative specimen in pancreatic head, showing a tumor composed of mixed cystic and solid components with hemorrhagic areas. White arrow demarcates region of pancreatic tail mass. PD pancreaticoduodenectomy, GB gallbladder, CBD common bile duct.

**Figure 3:**
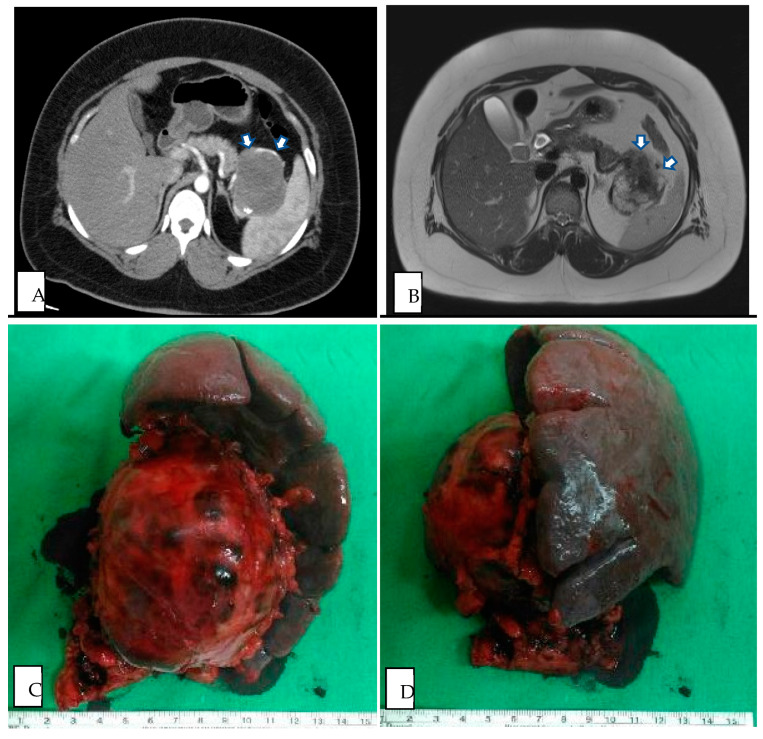
(**A**) Preoperative CT image of SPN in pancreatic tail (axial plane, venous phase). (**B**) Preoperative MRI image of SPN in pancreatic tail (axial plane, T2 weighted). (**C**) Intraoperative specimen of DP with splenectomy from SPN in pancreatic tail, ventral view. (**D**) Intraoperative specimen of DP with splenectomy from SPN in pancreatic tail, dorsal view. White arrow demarcates region of pancreatic tail mass.

All of our patients had preoperative radiologic findings in either US, CT, or MRI. Most showed typical features of SPN (Figure 2 and Figure 3), such as oval, exophytic, and regular capsulated lesions with a mixed cystic and solid component but were almost entirely solid or cystic with thick walls [7]. Preoperative imaging studies included US (10/24 cases), CT (23/24 cases), and MRI (14/24 cases). In total, nine cases were diagnosed as SPNs based on preoperative imaging, four cases based on CT (17%), and five cases based on MRI (36%) (Table 1). The CT modalities are shown in Table 2; all tumor margins were clear in preoperative images. No hemorrhage, parenchyma atrophy, or invasion of adjacent vessels and abdominal organs was seen. More irregular shaping was seen in SPNs of the pancreatic head (*p* = 0.037). The cystic component was significantly more prominent in the SPN of the pancreatic tail (*p* = 0.035). Pancreas or bile duct dilation was mostly seen in the pancreatic head in our patient (*p* < 0.001).

Preoperative pathological EUS fine needle biopsies were performed in 11 patients, with only 2 cases being preoperatively diagnosed as pancreatic SPNs (Table 1).

Of these 24 patients, 8 were diagnosed with pancreatic SPNs located at the pancreatic head, and 16 were diagnosed with pancreatic SPNs located at the distal pancreas (tail, 9 cases, 37.5%; body and tail, 2 cases 8.3%; body, 5 cases, 20.8%). We included the body and tail group and the tail group into the “body to tail group” for analysis between locations in Table 1.

### 3.3. Postoperative Outcomes and Complications

Among the patients with distal pancreatic SPNs, 13 underwent DP with splenectomy, 1 underwent DP without splenectomy, 1 underwent single port laparoscopic DP with splenectomy, 1 underwent robotic (Xi)-assisted CP and gastropancreaticostomy without splenectomy, and 1 underwent laparoscopic DP with splenectomy (Table 3). The median tumor size in the pathologic report was 7.6 (4.1–9.2) cm. Furthermore, a trend was identified that patients in the MI group had smaller tumor sizes (median, MI 3.1 [1.7–7.6] vs. conventional 7.8 [5.0–9.4] cm; *p* = 0.052).

No margin involvement was present in the pathologic reports of the enrolled patients. The immunohistochemistry (IHC) stains were mostly detected in CD10 (14 cases, 100% in CD10 stain cases), CD56 (7 cases, 100% in CD56 stain cases), and B-catenin (12 cases, 100% in B-catenin stain cases) (Table 4). Little positive immunolabeling was found in synaptophysin, chromogranin, vimentin, NSE, alpha-1-antichymotrypsin, and CyclinD1 but no strong correlation was detected. No histopathological correlation was found in prognoses or locations (Table 4).

Three complications were registered in the records: bile leakage and hepato-jejunostomy stricture requiring endoscopic balloon dilation after PPPD, postoperative pseudocyst after PPPD requiring no surgical intervention, and postoperative pancreatic tail hematoma after DP (body) with splenectomy, which subsided under conservative treatment. The cases included in this study were followed up till March 2023, with a median follow-up of 65.5 (34.5–116.5) months. The longest follow-up observed was 229 months after DP, and the patient had disease stability at the last visit. Further, 16 patients were lost during follow-up, and 6 patients had stable disease statuses at the end of our study.

A 38-year-old woman developed liver metastasis 41 months after undergoing PD, receiving chemotherapy once, and refusing subsequent doses due to severe side effects; she initiated palliative transcatheter arterial chemoembolization (TACE). Spleen metastasis and carcinomatosis were successively found, and she was lost during follow-up 10 years postoperatively.

A 9-year-old girl suffered from jaundice after PD, and nonalcoholic steatohepatitis (NASH) was diagnosed through liver biopsy 18 months after PD. She received living-related liver transplantation 70 months after the operation and passed away 115 months after PD due to graft failure related to psychological conditions and poor immunosuppressant adherence.

A 28-year-old woman, with a 7.6 cm tumor over the pancreatic tail, developed post pancreatectomy diabetes (1/27, 4.2%) in the follow-up period, after DP (body) with splenectomy.

During the follow-up period, two patient deaths occurred; one died after liver transplantation, as mentioned above, and the other died of colon cancer with liver and lung metastasis 12 months after receiving DP with splenectomy.

Statistically significant differences were identified between locations in terms of estimated blood loss (EBL), day of enteral feeding, and length of hospitalization in our study. No statistically significant differences were found between conventional and MI groups in terms of EBL, day of enteral feeding, day of drainage tube removal, length of hospitalization, follow-up period, post-operative pancreatic fistula grade, and delayed gastric emptying (DGE) grade in our study.

## 4. Discussion

In our single-center experience of pancreatic SPNs, only 12.5% of the patients were men in our population, which is consistent with another report, revealing that 10% of these tumors are diagnosed in men [5]. The median age of our patients was 38.0 (28.0–45.8) years in the conventional group and 23.5 (19.5–27.5) years in the MI group, without significant differences, as others have reported typically in the 2–3 decades of life [20]. The younger age in the MI group may be attributed to the progression of the health check concept in the modern world, the higher cosmetic demand in young populations, and better accessibility to image examinations.

Patients in our study underwent conventional surgery or MI surgery for SPNs, showing no statistically significant differences in terms of sex, age, BMI, and perioperative outcomes, which are similar to the findings of previous reports on pancreatic SPN management [21,22]. Most symptoms of our patients were nonspecific, and diagnosis was incidentally encountered through imaging examinations. Other symptoms included abdominal pain (eight cases, 29%), palpable mass lesions (four cases, 17%), and tarry stool (one case, 4.2%), which aligns with previous research [5,10,20,21]. Tumor markers, such as carbohydrate antigen (CA) 19-9 and carcinoembryonic antigen, were almost within the normal range, as previous studies have reported [4,10,13,21].

CT is the most commonly used preoperative imaging study and plays a significant role in the diagnosis of cystic lesions of the pancreas due to its cost-effectiveness in detecting and characterizing pancreatic SPNs, while MRI may allow for better identification of several tissue characteristics, such as hemorrhage, cystic degeneration, or the presence of a capsule [10,20,23,24]. If MRI reveals a well-marginated, encapsulated, solid, and cystic mass, with areas of hemorrhagic degeneration, a diagnosis of pancreatic SPN should be considered [23]. The role of EUS fine needle biopsy in pancreatic SPNs is not entirely established due to a lack of precise preoperative pathological diagnoses (diagnosis ratio: 2/11, 18.18% in our study). Furthermore, EUS fine needle biopsy can also lead to complications, such as rupture and peritoneal seeding, according to a previous study [11]. According to our experience, EUS fine needle biopsy was applied more frequently to exclude other malignant pancreatic lesions than to confirm pancreatic SPN diagnosis. Research has also been conducted on the non-invasive detection of pancreatic cancer utilizing fecal microbiota signatures in conjunction with serum levels of CA19-9 [25,26]. This method could potentially improve our preoperative differentiation between SPNs and pancreatic cancer.

The most common location of SPNs in our study was the pancreatic tail (nine cases, 37.5%), with other locations including the pancreatic head (eight cases, 33.3%), body (five cases, 21%), and body and tail (two cases, 8.3%), which is similar to those reported by Kang and Uğuz [14,27]. In image modalities, we found more irregular shapes seen in the pancreatic head (*p* = 0.037) and more cystic components in the pancreatic tail (*p* = 0.035), which were not well established in previous research. We may find better correlations in image modalities based on locations for better preoperative diagnoses with greater data collection. Pancreas or bile duct dilation was mostly seen in SPNs of the pancreatic head (*p* < 0.001), which may be related to the pancreas anatomy.

We also found an increasing incidence of SPN in the past 10 years in our patient group (10 patients before 2010 and 14 patients after 2010), which may be due to an increase in the health check-up concept, imaging studies, EUS fine needle aspiration, and pathology findings, as reported by Law et al. [9].

The statistical significance in postoperative outcome comparisons between locations may result from the different complexities of anatomy between PD (including PD and PPPD) and DP (including CP and partial pancreatectomy).

The smaller size of the MI surgery group (median, 3.1 [1.7–7.6] vs. 7.8 [5.0–9.4] cm) may be due to the advances in imaging modalities that lead to higher accuracy in the diagnosis of pancreatic SPN, as reported by Machado et al., who showed that pancreatic SPNs detected after 2000 were smaller than those diagnosed before [28]. However, the smaller tumor size in the MI group may have been a factor in deciding which surgical approach to follow, as previous studies have reported [16,29].

Our data also revealed a trend of an increased ratio of MI surgery (0 MI procedures in 10 cases before 2010; 4 MI surgeries out of 14 [29%] after 2010), which may be due to the progress of MI surgery skills, MI instruments, and the robotic system, as published by Cawich et al. [30]. However, the SPN locations ratio was similar before and after 2010 (head/body/tail; before 3/3/4, after 5/4/5).

No residual tumor was found after the operation, and all pathological reports indicated margin-free statuses in all cases in our study. This result suggests that MI and conventional operations deliver comparable long-term oncological results, as reported by Tan et al. [29]. Although some of the literature has discussed circumferential resection margins (CRM) in pathology reports of pancreatic cancer, there was no consensus on CRM for SPNs in our hospital. Patient enrollment commenced in 1998, and the perspective on CRM was introduced in 2006. Moreover, CRM was not addressed in earlier studies of SPNs due to the generally positive oncological outcomes associated with this condition [31,32].

The most commonly detected immunohistochemistry (IHC) stains in our study included CD10 (14 cases, 100% in CD10 stain cases), CD56 (7 cases, 100% in CD56 stain cases), and B-catenin (12 cases, 100% in B-catenin stain cases). As reported by Watanabe et al., vimentin, CD10, and CD56, are characteristically positive, and B-catenin was involved in the pathogenesis of SPN [6]. However, the histopathological features demonstrated no correlations in locations and prognoses in our study, like other reports [33].

In one case (4.2%), liver metastasis was found 41 months postoperatively, which matched the 2–10% postoperative recurrence after SPN radical resection reported previously [34]. Previous research has also indicated that the liver was the most common site of distant metastasis [21,35]. Despite the chemotherapy and transarterial chemoembolization (TACE) treatments that the patient received after liver metastasis, carcinomatosis still developed during the follow-up period, consistent with many reports showing a limited response to different regimens of chemotherapy [4,13,21]. Nevertheless, long-term survival was still observed; the patient survived for 159 months after surgery. This highlights the good oncological prognosis of SPN after surgery, which has been reported to range from 7 to 10 years after undergoing complete resection, even in patients with residual and disseminated diseases [10,13,21]. Sumida et al. reported that living donor liver transplantation for SPN with multiple liver metastatic lesions after complete resection was a possible therapeutic procedure with a >2 years of disease-free survival [35].

A 9-year-old female patient in our study suffered from jaundice after PD, and NASH was diagnosed through liver biopsy 18 months after PD. Though pediatric pancreatic tumors and PD in pediatric patients are rare, Sawai et al. reported a 10-year-old girl suffering from nonalcoholic fatty liver disease (NAFLD) developed after PD for SPN [36]. No previous reports describe NAFLD in children after PD due to its rarity. The mechanism of NAFLD or NASH remains uncertain, with a few reports hypothesizing that decreased exocrine function, deficiency in zinc, or bacterial translocation due to intestinal mucosal atrophy after PD play a role [36]. McGhee-Jez et al. reported an increased risk of post-PD NAFLD in women and adult patients with pancreatic cancer, shorter postoperative hospital stays, or higher preoperative BMIs [37]. Though the role of post-PD pancreatic enzyme supplementation is under debate, closely following patient status and pancreatic enzyme supplementation are suggested in pediatric patients after PD according to our experience [36,37]. We should also place more emphasis on psychological care in young patients who undergo pancreatic surgery.

Our data included a case of post-pancreatectomy diabetes in a patient who was at risk due to an extremely high BMI (43.75), as reported by Kwon JH. [38]. In our study, endocrine function was well preserved, even under aggressive resection (post-pancreatectomy diabetes, 4.2%), similar to other reports [39,40]. Some research reports 10–20% exocrine insufficiency after pancreatectomy, with steatorrhea or weight loss resolving after pancreatic enzyme supplementation [39,40].

According to the parenchyma preserving concept, we performed robotic (Xi)-assisted CP and gastropancreaticostomy with spleen preservation in a 26-year-old woman with a 3.2 cm tumor over the pancreatic body; no specific postoperative complications developed. No recurrence nor distant metastasis were found during the 17-month follow-up period. In our study, we observed no steatorrhea nor weight loss after pancreatectomy during the follow-up period. Despite some reports suggesting a parenchyma-preserving approach for SPN, due to its low malignant risk and possible decline in pancreatic endocrine and exocrine functions, oncologic outcomes were unclear due to the incremental risk of margin involvement. In our experience, parenchyma-preserving operations may be safe and feasible for treating pancreatic SPNs, but further research is required to clarify the benefits and drawbacks of parenchyma-preserving operations [21,27,39,40].

Our study has several limitations. Firstly, a notable challenge lies in the scarcity of high-quality, prospective, randomized controlled studies investigating the role of MI surgery in pancreatic SPN, largely due to its rare epidemiology. To address this gap, further studies are warranted, such as multi-center surveys or studies involving larger patient cohorts. These studies should aim to compare the efficacy of various surgical approaches through block or stratified randomization, thereby facilitating the acquisition of high-level evidence regarding the optimal management strategies for SPN.

Secondly, it is notable that our hospital lacks standardized preoperative evaluation protocols, encompassing diabetes profiles, imaging studies, and EUS reports. Particularly significant is the absence of a standardized immunohistochemistry stain in our hospital, considering the pathological variations identified in the research on SPN over the extended study period. Furthermore, our hospital lacks a standardized follow-up protocol for postoperative outcomes, including imaging, follow-up schedules, and diabetes profiles.

Thirdly, the extensive study period spanning more than 20 years poses challenges in evaluating not only the surgical techniques but also preoperative radiological diagnostics. This is particularly attributable to the advancements in MI techniques, MI equipment, and radiological facilities observed over this prolonged duration.

## 5. Conclusions

In conclusion, aggressive complete resection of pancreatic SPNs can be suggested based on our experience and according to the promising long-term survival achieved. Furthermore, this operation leads to no more complications than conventional surgery in treating SPNs of the pancreatic head and distal locations with good oncological outcomes. No histopathological correlations were found in prognoses and locations.

## Figures and Tables

**Table 1:** Patient characteristics and surgical outcomes between different locations (n = 24).

	Total (n = 24)		Head (n = 8)		Body (n = 5)		* Body to Tail (n = 11)		*p*-Value
Age	34.5	(23.8–41.5)	30.5	(18.0–45.8)	28	(21.5–56)	38	(28.0–40.0)	0.596
Sex									1.000
Female	21	(87.5%)	7	(87.5%)	4	(80%)	10	(91%)	
Male	3	(12.5%)	1	(12.5%)	1	(20%)	1	(9%)	
BMI	20.8	(18.9–22.4)	19.0	(18.3–23.4)	21.1	(18.8–21.9)	21.1	(20.4–22.9)	0.561
Size	7.6	(4.1–9.2)	6.0	(4.1–9.9)	3.2	(1.6–7.5)	9.0	(7.5–9.3)	0.149
CEA	1.5	(1.1–3.3)	1.6	(1.1–3.3)	1.5	(1.1–3.1)	1.5	(1.1–3.4)	0.992
CA19-9	12.8	(5.7–29.1)	10.8	(5.0–19.4)	26.8	(8.3–457.8)	12.8	(5.3–29.9)	0.467
Preop diagnosis									
US	10	(41.7%)	3	(37.5%)	2	(40%)	5	(46%)	1.000
CT	23	(95.8%)	8	(100%)	5	(100%)	10	(91%)	0.540
SPN in CT	4/23	(17.4%)	1/8	(12.5%)	0/5	(0%)	3/10	(30%)	0.368
MRI	14	(58.3%)	7	(87.5%)	4	(80%)	3	(27%)	0.021 *
SPN in MRI	5/14	(35.7%)	2/7	(28.6%)	2/4	(50%)	1/3	(33%)	0.790
EUS	11	(45.8%)	5	(62.5%)	4	(80%)	2	(18%)	0.047 *
SPN in EUS	2/11	(18.2%)	1/5	(20.0%)	1/4	(25%)	0	(0%)	1.000
Operations									
Conventional	20	(83.3%)	7	(87.5%)	3	(60%)	10	(89%)	
PD or PPPD	7	(29%)	7	(0%)	0	(0%)	0	(0%)	
DP	13	(48%)	0	(0%)	3	(60%)	10	(89%)	
CP	0	(0%)	0	(0%)	0	(0%)	0	(0%)	
MI	4	(16.7%)	1	(12.5%)	2	(40%)	1	(11%)	
PD or PPPD	1	(4%)	1	(12.5%)	0	(0%)	0	(0%)	
DP	2	(8%)	0	(0%)	1	(20%)	1	(11%)	
CP	1	(4%)	0	(0%)	1	(20%)	0	(0%)	
Complications									
EBL, mL	200	(50–665)	380	(212.5–803.8)	50	(50–125)	260	(50–738)	0.028 *
EF, days	4	(3–5)	5	(4–8)	4	(2–5)	3.5	(3–4)	0.017 *
RD, days	11	(6–22)	18	(7–20)	13	(5–25.5)	7	(6–26)	0.980
LoH, days	10	(7–21)	21	(12–27)	8	(7–11.5)	7.5	(7–13)	0.010 *
F/U, months	65.5	(34.5–116.5)	99	(61.5–139)	41	(9–108)	52	(26–99)	0.248
Leakage, ISPGF									0.432
N	10	(43.5%)	4	(50.0%)	1	(20.0%)	5	(50%)	
A	6	(26.1%)	3	(37.5%)	2	(40.0%)	1	(10%)	
B	7	(30.4%)	1	(12.5%)	2	(40.0%)	4	(40%)	
DGE, ISPGS									0.083
N	18	(81.8%)	4	(57.1%)	4	(80.0%)	10	(100%)	
A	2	(9.1%)	1	(14.3%)	1	(20.0%)	0	(0%)	
B	2	(9.1%)	2	(28.6%)	0	(0%)	0	(0%)	

* Chi-square test or Mann–Whitney U test, median (IQR); * *p* < 0.05. Body to tail group included the body and tail and the tail group. DP distal pancreatectomy, CP central pancreatectomy, PD Whipple operation, PPPD pylorus-preserving pancreaticoduodenectomy, MI minimal invasive, BMI body mass index, CEA carcinoembryonic antigen, CA19-9 carbohydrate antigen 19-9, US ultrasonography, CT computed tomography, SPN solid pseudopapillary neoplasm, MRI magnetic resonance imaging, EUS endoscopic ultrasonography, EBL estimated blood loss, RD remove drainage, LoH length of hospitalization, SPN solid pseudopapillary neoplasm, ISGPF International Study Group on Pancreatic Fistula, DGE delayed gastric emptying, ISGPS International Study Group of Pancreatic Surgery.

**Table 2:** Computed tomography characteristics in our patients.

	Total (n = 24)		Head (n = 8)		Body (n = 5)		* Body to Tail (n = 11)		*p*-Value
Shape									0.037 *
Oval	20	(87.0%)	5	(62.5%)	5	(100.0%)	10	(100.0%)	
Irregular	3	(13.0%)	3	(37.5%)	0	(0.0%)	0	(0.0%)	
Ratio of solid-to-cystic components									0.035 *
Solid	9	(39.1%)	4	(50.0%)	3	(60.0%)	2	(20.0%)	
Half	6	(26.1%)	4	(50.0%)	0	(0.0%)	2	(20.0%)	
Cystic	8	(34.8%)	0	(0.0%)	2	(40.0%)	6	(60.0%)	
Capsule complete									0.308
N	2	(8.7%)	1	(12.5%)	1	(20.0%)	0	(0.0%)	
Y	21	(91.3%)	7	(87.5%)	4	(80.0%)	10	(100.0%)	
Margin clear									--
N	0	(0.0%)	0	(0.0%)	0	(0.0%)	0	(0.0%)	
Y	23	(100.0%)	8	(100.0%)	5	(100.0%)	10	(100.0%)	
Growth pattern									0.082
Exophytic	20	(87.0%)	7	(87.5%)	3	(60.0%)	10	(100.0%)	
Intra	3	(13.0%)	1	(12.5%)	2	(40.0%)	0	(0.0%)	
Margin calcification									0.663
n	14	(60.9%)	6	(75.0%)	3	(60.0%)	5	(50.0%)	
y	9	(39.1%)	2	(25.0%)	2	(40.0%)	5	(50.0%)	
Hemorrhage									--
n	23	(100.0%)	8	(100.0%)	5	(100.0%)	10	(100.0%)	
y	0	(0.0%)	0	(0.0%)	0	(0.0%)	0	(0.0%)	
Compression of the main pancreatic duct and bile duct									0.565
n	22	(95.7%)	7	(87.5%)	5	(100.0%)	10	(100.0%)	
y	1	(4.3%)	1	(12.5%)	0	(0.0%)	0	(0.0%)	
Upstream pancreatic parenchymal atrophy									--
n	23	(100.0%)	8	(100.0%)	5	(100.0%)	10	(100.0%)	
y	0	(0.0%)	0	(0.0%)	0	(0.0%)	0	(0.0%)	
Invasion of adjacent vessels and abdominal organs									--
n	23	(100.0%)	8	(100.0%)	5	(100.0%)	10	(100.0%)	
y	0	(0.0%)	0	(0.0%)	0	(0.0%)	0	(0.0%)	
Pancreas or bile duct dilation									<0.001 **
n	15	(65.2%)	1	(12.5%)	4	(80.0%)	10	(100.0%)	
Dilate	8	(34.8%)	7	(87.5%)	1	(20.0%)	0	(0.0%)	

Fisher’s exact test. * *p* < 0.05, ** *p* < 0.01. There was one patient without any pre-operative image study. Body to tail group included the body and tail and the tail group intra intrapancreatic.

**Table 3:** Procedure list.

	Procedure	Number
Conventional	PD	3
	PPPD	4
	DP (body) with splenectomy	10
	DP (body) with splenectomy and stomach wedge resection	1
	DP (body) with spleen preservation	1
	DP (tail) with splenectomy and radical nephrectomy	1
Minimally invasive	Robotic (Si) assisted PPPD	1
	Robotic (Xi) assisted CP and gastropancreaticostomy with spleen preservation	1
	Single port laparoscopic DP (tail) with spleen preservation	1
	Laparoscopic DP (Tail)	1

Si Da Vinci Si surgical system, Xi Da Vinci Xi surgical system, PD pancreaticoduodenectomy, PPPD Pylorus preserving pancreaticoduodenectomy, DP distal pancreatectomy, CP central pancreatectomy.

**Table 4:** Histopathological profiles of SPNs in our patients.

	Total (n = 24)		Head (n = 8)		Body (n = 5)		* Body to Tail (n = 11)		*p*-Value
B-catenin									1.000
NT	12	(50.0%)	4	(50.0%)	3	(60.0%)	5	(45.5%)	
P	12	(50.0%)	4	(50.0%)	2	(40.0%)	6	(54.5%)	
N	0	(0.0%)	0	(0.0%)	0	(0.0%)	0	(0.0%)	
CD10									1.000
NT	8	(33.3%)	3	(37.5%)	1	(20.0%)	4	(36.4%)	
P	16	(66.7%)	5	(62.5%)	4	(80.0%)	7	(63.6%)	
N	0	(0.0%)	0	(0.0%)	0	(0.0%)	0	(0.0%)	
CD56									1.000
NT	2	(8.7%)	1	(12.5%)	1	(20.0%)	0	(0.0%)	
P	21	(91.3%)	7	(87.5%)	4	(80.0%)	10	(100.0%)	
N	0	(0.0%)	0	(0.0%)	0	(0.0%)	0	(0.0%)	
Chromatin									0.310
NT	10	(41.7%)	4	(50.0%)	0	(0.0%)	6	(54.5%)	
P	6	(25.0%)	2	(25.0%)	2	(40.0%)	2	(18.2%)	
N	8	(33.3%)	2	(25.0%)	3	(60.0%)	3	(27.3%)	
Synaptophysin									0.153
NT	20	(83.3%)	7	(87.5%)	3	(60.0%)	10	(90.9%)	
P	3	(12.5%)	0	(0.0%)	2	(40.0%)	1	(9.1%)	
N	1	(4.2%)	1	(12.5%)	0	(0.0%)	0	(0.0%)	
Cyclin D1									0.803
NT	20	(83.3%)	6	(75.0%)	4	(80.0%)	10	(90.9%)	
P	3	(12.5%)	1	(12.5%)	1	(20.0%)	1	(9.1%)	
N	1	(4.2%)	1	(12.5%)	0	(0.0%)	0	(0.0%)	
Vimentin									1.000
NT	16	(66.7%)	6	(75.0%)	3	(60.0%)	7	(63.6%)	
P	8	(33.3%)	2	(25.0%)	2	(40.0%)	4	(36.4%)	
N	0	(0.0%)	0	(0.0%)	0	(0.0%)	0	(0.0%)	
PR									0.501
NT	16	(66.7%)	4	(50.0%)	4	(80.0%)	8	(72.7%)	
P	6	(25.0%)	2	(25.0%)	1	(20.0%)	3	(27.3%)	
N	2	(8.3%)	2	(25.0%)	0	(0.0%)	0	(0.0%)	

Fisher’s exact test. * *p* < 0.05. Body to tail group included the body and tail and the tail group. NT not tested, P positive, N negative, SPN, solid pseudopapillary neoplasm.

## Data Availability

Data are unavailable due to privacy or ethical restrictions.
